# Study on the Biochemical Characterization and Selectivity of Three β-Glucosidases From *Bifidobacterium adolescentis* ATCC15703

**DOI:** 10.3389/fmicb.2022.860014

**Published:** 2022-04-08

**Authors:** Yanbo Hu, Liyuan Zhai, Huili Hong, Zenghui Shi, Jun Zhao, Duo Liu

**Affiliations:** ^1^School of Food Science and Engineering, Changchun University, Changchun, China; ^2^School of Life Sciences, Changchun Normal University, Changchun, China

**Keywords:** β-glucosidase, *Bifidobacterium adolescentis*, biochemical characterization, selectivity, biotransformation

## Abstract

Three β-glucosidases from *Bifidobacterium adolescentis* ATCC15703, namely, BaBgl1A, BaBgl3A, and BaBgl3B, were overexpressed in *Escherichia coli*. The recombinant β-glucosidases were sufficiently purified using Ni^2+^ affinity chromatography, and BaBgl1A exhibited the best purification efficiency with a purification factor of 2.3-fold and specific activity of 71.2 U/mg. Three recombinant β-glucosidases acted on *p*-nitrophenyl-β-glucopyranoside (*p*NPβGlc) at around pH 7.0 and 30–50°C. The results of the substrate specificity assay suggested that BaBgl1A acted exclusively as β-1,2-glucosidase, while BaBgl3A and BaBgl3B acted mostly as β-1,3-glucosidase and β-1,4-glucosidase, respectively. The substrate specificity of the three recombinant enzymes was further studied using the ginsenosides Rb1 and Rd as substrates. The results of thin-layer chromatography and high-performance liquid chromatography analyses showed that BaBgl1A exhibited the highest bioconversion ability on Rb1 and Rd, where it hydrolyzed the outer C-3 glucose moieties of Rb1 and Rd into the rare ginsenosides Gypenoside XVII and F_2_; BaBgl3A exhibited medium bioconversion ability on Rb1, where it hydrolyzed both the outer C-3 and C-20 glucose moieties of Rb1 into Gyp XVII and Rd; and BaBgl3B was not active on Rb1 and Rd. These β-glucosidases will act as new biocatalytic tools for transforming ginsenosides and preparing active glycosides and aglycone.

## Introduction

Glycoside hydrolases, which hydrolyze and rearrange glycosidic bonds, are found ubiquitously in bacteria, fungi, plant seeds, and animals (Scigelova et al., 1999). At present, 172 glycoside hydrolase families have been reported based on sequence and structural similarities in the CAZy database (Toma et al., 2021). As the main component of glycoside hydrolases, β-glucosidases (BGLs, EC 3.2.1.21) are classified into families GH1, GH2, GH3, GH5, GH9, GH30, GH39, and GH116, which hydrolyze the terminal, non-reducing β-D-glucosyl residues to release β-D-glucose. So far, GH1 is known to have the largest number of known β-glucosidase members with the highest diversity of substrate specificities (Chinchetru et al., 1989; Cantarel et al., 2009).

*Bifidobacterium* is an important human intestinal probiotic, which can hydrolyze and metabolize oligosaccharides and produce small molecules beneficial for the human body, such as lactate, acetate, short-chain fatty acids, and propionate (Saraoui et al., 2013; Louis et al., 2014). The glycoside hydrolases play an important role in the metabolism of these oligosaccharides. Those glycosidases mainly included β-glucosidases, β-galactosidase, α-glucosidase, and α-galactosidase (Kenji et al., 1986; Xiao et al., 2000; Hsu et al., 2007; Kim et al., 2017). β-Glucosidase, the key enzyme required for the metabolism and homeostasis of *Bifidobacterium*, can hydrolyze oligosaccharides to produce glucose, which can be further used as an energy source of organisms (Audrey et al., 2016). Therefore, the biochemical characterization and determination of the selectivity of those β-glucosidases will be helpful for understanding the molecular mechanism of oligosaccharide degradation by probiotics and developing new prebiotics. The ways to obtain those β-glucosidases from *Bifidobacterium* mainly involved purification and recombinant expression. Two β-glucosidases have been successfully purified from *Bifidobacterium breve* 203, and they exhibited a different activity after the addition of glucose (Kenji et al., 1986). Furthermore, the β-D-fucosylglucose activity of β-D-glucosidase I was enhanced in the mutagenic strain *Bifidobacterium breve* clb (Sakai et al., 1989). Biotransformation by recombinant β-glucosidases is better than that by native β-glucosidases due to their high yield and stability during the biotransformation process (Shin and Oh, 2014). Certain β-glucosidases from *Bifidobacterium*, such as *Bifidobacterium longum* subsp. Infantis ATCC15697 (Matsumoto et al., 2015), *Bifidobacterium adolescentis* ATCC15703 (Florindo et al., 2018), *Bifidobacterium breve* 203 (Nunoura et al., 1997), and *Bifidobacterium pseudocatenulatum* IPLA 36007 (Guadamuro et al., 2017), can be expressed in *Escherichia coli* as soluble proteins. There were also other expression systems for the preparation of recombinant β-glucosidase, such as the β-glucosidase gene from *Bifidobacterium animalis* subsp. Lactis, which was overexpressed in *Bifidobacterium bifidum* BGN4 (Youn et al., 2012). However, these β-glucosidases were not substrate specific, as some of them exhibited other hydrolyzing activities in addition to the hydrolysis of *p*-nitrophenyl-β-glucopyranoside (*p*NPβGlc). Therefore, the identification and utilization of novel and specific β-glucosidase will be useful for understanding the molecular mechanism of oligosaccharide degradation by probiotics.

In this study, we have biochemically characterized three novel β-glucosidases (BaBgl1A, BaBgl3A, and BaBgl3B) from *B. adolescentis* ATCC15703. The results showed that the three β-glucosidases exhibited a multifunctional hydrolyzing activity toward different glycosides and aglycones. The biochemical characterization and selectivity of those β-glucosidases will be helpful to understand the molecular mechanism of oligosaccharide degradation by probiotics and to develop new prebiotics.

## Materials and Methods

### Bacteria and Reagents

*Bifidobacterium adolescentis* ATCC15703, isolated from the intestine of an adult, was purchased from BeNa Culture Collection. The p-nitrophenyl glycoside derivatives, including *p*-nitrophenyl-β-D-glucopyranoside (*p*NPβGlc), *p*-nitrophenyl-α-D-glucopyranoside (*p*NPαGlc), *p*-nitrophenyl-β-D-galactopyranoside (*p*NPβGal), *p*-nitrophenyl-α-D-galact opyranoside (*p*NPαGal), *p*-nitrophenyl-β-D-xylopyranoside (*p*NPβXyl), *p*-nitrophenyl-α-D-xylopyranoside (*p*NPαXyl), *p*-nitrophenyl-α-L-arabinofuranoside (*p*NPαAra*f*), and *p*-nitro phenyl-α-L-arabinopyranoside (*p*NPαAra*p*), were purchased from Yuanye Bio-Technology Co., Ltd. (Shanghai, China). The other chemicals, including sophorose, laminaribiose, cellobiose, gentiobiose, Rb1, and Rd, were purchased from Solarbio Science & Technology Co., Ltd. (Beijing, China).

### Gene Cloning and Protein Expression

The coding region of the genes encoding the three β-glucosidases was amplified from the genomic DNA of *B. adolescentis* ATCC15703 using polymerase chain reaction with the primers shown in Table 1 and cloned into the *p*ET-28a vector. The recombinant plasmids were sent to Comate Bioscience Co., Ltd., for sequencing. The resulting constructs of *pet-28a-babgl1a*, *pet-28a-babgl3b*, and *pet-28a-babgl1a* were transformed into *E. coli* BL21 (DE3) and grown in Luria–Bertani medium. The expression of the target protein was induced with 0.5 mM isopropyl β-D-1-thiogalactopyranoside at 16°C for 20 h. The recombinant protein was purified using Ni-Sepharose 6 Fast Flow affinity chromatography as described previously (Zhu et al., 2015). The enzyme purity and molecular mass were determined using sodium dodecyl sulfate-polyacrylamide gel electrophoresis, and the gels were both stained with Coomassie Brilliant Blue R250 (Conway et al., 2016) to visualize proteins. The protein concentration was determined using bovine serum albumin as the standard and the Coomassie Brilliant Blue method (Bradford, 1976).

**TABLE 1:** Summary of cloned glucosidase genes from *Bifidobacterium adolescentis* ATCC15703.

Name	Gene names	Primers	
		
		Orientation	Sequence (5–3)
BaBgl1A	BAD_1197	Forward	GACTGGTGGACAGCAAATGGGTCGCGGATCCATGAAAGAACAATACGAGT
		Reverse	ATCTCAGTGGTGGTGGTGGTGGTGCTCGAGCTATCTGGCCATGACCCCCT
BaBgl3A	BAD_1194	Forward	GACTGGTGGACAGCAAATGGGTCGCGGATCCATGAGCGAAAACACCTATC
		Reverse	ATCTCAGTGGTGGTGGTGGTGGTGCTCGAGTTATTCGGCGGTTTCGGCGA
BaBgl3B	BAD_1287	Forward	GACTGGTGGACAGCAAATGGGTCGCGGATCCATGTCGAGTTGCGGATGTG
		Reverse	ATCTCAGTGGTGGTGGTGGTGGTGCTCGAGTTACGCGACGGTGAAGGTGG

*The underline terms means restriction enzyme cutting site.*

### Enzyme Activity Assay

The enzyme activity of the three β-glucosidases was determined by measuring the hydrolysis of the substrate *p*NPβGlc (30 mM) (Strahsburger et al., 2017). The reaction mixture of 200 μl, composed of 10 μl β-glucosidases (0.3 mg/ml), 20 μl *p*NPβGlc (30 mM), and 170 μl buffer (pH 7.0), was incubated at 37°C for 10 min. Then, the reaction was terminated by adding 50 μl Na_2_CO_3_ (0.5 M). The amount of *p*-nitrophenol (*p*NP) liberated from *p*NPβGlc was determined by measuring the absorption at 405 nm by BioTek ELx808 microplate reader (Winooski, VT, United States). The calculation of enzyme activity was based on *p*NP standard curve. One unit (U) of β-glucosidase activity was defined as the amount of enzyme that released 1 μmol of *p*NP per minute under the assay conditions.

### Enzymatic Properties

The optimum pH was determined by monitoring the enzyme activity with *p*NPβGlc at different pH conditions (Dan et al., 2020). The optimal pH of the enzyme for *p*NPβGlc hydrolysis was determined at 37°C and pH 3.5–11.0 using 50 mM NaAc (pH 3.5–6.0), Na_2_HPO_4_–NaH_2_PO_4_ (pH 6.0–8.0), and Gly-NaOH (pH 8.0–11.0) buffers. A total of 200 μl of the reaction mixture containing 10 μl β-glucosidases (0.3 mg/ml), 20 μl *p*NPβGlc (30 mM), and 170 μl of different buffers was incubated at 37°C for 10 min. The reaction mixture without enzyme was used as a blank, and then the reaction was terminated by adding 50 μl Na_2_CO_3_ (0.5 M). The pH stability was determined by measuring the residual enzyme activity after incubation in buffers with varying pH at 4°C for 12 h.

The optimum temperature was determined by measuring the enzyme activity at optimal pH in the temperature range of 20–70°C. A total of 200 μl of the reaction mixture containing 10 μl β-glucosidases (0.3 mg/ml), 20 μl *p*NPβGlc (30 mM), and 170 μl optimal buffers was incubated at different temperatures for 10 min. The reaction mixture without enzyme was used as a blank, and then the reaction was terminated by adding 50 μl Na_2_CO_3_ (0.5 M). The thermal stability of BaBgl1A was determined by measuring the residual enzyme activity after incubation at 25–45°C for up to 2 h, with sampling after every 20 min. The thermal stabilities of BaBgl3A and BaBgl3B were determined by measuring the residual enzyme activity after incubation at 35–55°C for up to 2 h, with sampling after every 20 min.

### Effect of Additives on Enzyme Stability

The stability of the three β-glucosidases, when coincubated with factors that potentially affect enzyme activity, was evaluated (Jiang et al., 2021). Metal ions and other chemicals, including NaCl, KCl, MgCl_2_, CaCl_2_, CuCl_2_, BaCl_2_, HgCl, MnCl_2_, FeCl_2_, ethylenediaminetetraacetic acid (EDTA), and sodium dodecyl sulfate, were studied in this work. The enzymatic activities were tested in the presence of 5 or 25 mM (final concentration) of metal ions or other chemicals for 20 min at optimum pH and temperature. The residual activity of the three β-glucosidases was determined using *p*NPβGlc as a substrate as described before, and the activities are expressed as a percentage of the activity obtained in the absence of the compound. The effect of polyols on three β-glucosidases when incubated with the substrate in the presence of 2 M polyols, including glucose, xylose, mannose, galactose, arabinose, and fructose, was determined. For assessing the residual activity, the enzyme was initially incubated at optimum pH and temperature for 20 min. Then, the residual activity was determined at optimum temperature using *p*NPβGlc as substrate. Enzyme activity without any additive was included as the control (100%).

### Substrate Specificity

Substrate specificity was determined by measuring the enzyme activity on different artificial substrates (*p*NPβGlc, *p*NPαGlc, *p*NPβGal, *p*NPαGal, *p*NPβXyl, *p*NPαXyl, *p*NPαAra*f*, and *p*NPαAra*p*) and disaccharides (sophorose, laminaribiose, cellobiose, and gentiobiose). The reaction mixture of 200 μl, which was composed of 10 μl β-glucosidases (0.3 mg/ml), 20 μl of different artificial substrates (30 mM), and 170 μl of optimal buffers, was incubated at optimal temperature for 10 min. The reaction mixture without enzyme was used as a blank. The reaction was stopped by the addition of 50 μl Na_2_CO_3_ (0.5 M). For artificial substrates, the absorbance of the mixture was measured at 405 nm. The activities over disaccharides were measured following the 3,5-dinitrosalicylic acid method described previously (Zhu et al., 2016).

### Bioconversion of Ginsenosides Using β-Glucosidases

To investigate the bioconversion ability of the β-glucosidases derived from *B. adolescentis*, two different ginsenosides (Rb1 and Rd) were used as substrates. The enzyme (final concentration of 0.1 mg/ml in 50 mM buffer of optimum pH) was allowed to react with 5 mg/ml of ginsenosides (Rb1 and Rd) at 37°C for 2 h. The reaction was terminated by boiling the sample for 10 min to remove protein. Samples were taken, filtered, and analyzed using thin-layer chromatography (TLC) and-high performance liquid chromatography (HPLC). For TLC, GF_254_ silica gel plates were used, which were developed in a solvent system consisting of butyl alcohol/ethyl acetate/water (4:4:1, v/v/v) (Zheng et al., 2021). The spots on the TLC plates were visualized by spraying with 5% (v/v) H_2_SO_4_ and identified by comparing with the ginsenoside standards. A Shimadzu HPLC system (CTO-20A pump and SPD-20AVD UV detector) was used for the analysis. The separation was performed on a Unitary-C18 column (5 μm, 4.6 mm × 250 mm). The mobile phase consisted of water (solvent A) and acetonitrile (solvent B). The elution gradient consisted of 70% solvent A, followed by 100% solvent B for 0–70 min. The flow rate was 0.4–0.8 ml/min, the injection volume was 20 μl, and the detection wavelength was 203 nm (Kim et al., 2022).

## Results

### Physicochemical Characteristics of Recombinant β-Glucosidases

The sequence of the three β-glucosidases—BaBgl1A, BaBgl3A, and BaBgl3B—from *B. adolescentis* ATCC15703 (with GenBank accession numbers BAF40068.1, BAF39975.1, and BAF39978.1) contains 391, 751, and 809 amino acids with a theoretical molecular mass of 44.2, 81.1, and 87.8 kDa, respectively. The deduced amino acid sequence of BaBgl1A was similar to that of the GH1 family. The multiple amino acid sequence alignment indicated that BaBgl1A exhibited identities with some characterized GH1 β-glucosidases, such as β-glucosidases from *Alicyclobacillus acidiphilus* (45%), *Exiguobacterium antarcticu*m B7 (44%), and *Clostridium cellulovorans* (41%) (Jeng et al., 2011; Zanphorlin et al., 2016; Delgado et al., 2021). While the deduced amino acid sequences of BaBgl3A and BaBgl3B were similar to those of the GH3 family (CAZy database)^1^, the multiple amino acid sequence alignment indicated that BaBgl3A and BaBgl3B exhibited identities with some characterized GH3 β-glucosidases, including β-glucosidases from *Bacteroides ovatus*, *Bifidobacterium longum* subsp. longum KACC 91563, and *Listeria innocua* Clip11262 (Masahiro et al., 2016; Rikuto et al., 2017; Yan et al., 2018). Signal sequences were absent in the three β-glucosidases. The multiple amino acid sequence alignment indicated that the amino acid sequences of the three recombinant β-glucosidases were similar (Supplementary Figure 1), such as Phe71, Pro72, Ala92, Glu128, and Asp129. These amino acids might be present in the main domain required for glucosidase activity. The structure of these β-glucosidases was evaluated by PyMOL software. The results indicated that the active sites of BaBgl1A were Glu159 and Glu306, while the active sites of BaBgl3A and BaBgl3B were both composed of Asp and Glu, the sites of BaBgl3A were Asp232 and Glu417, and the sites of BaBgl3A was Asp306 and Glu549. In addition, BaBgl3A and BaBgl3B were more similar to each other than BaBgl1A, with a similarity level of 32%. This may be because both BaBgl3A and BaBgl3B were members of the GH3 family, while BaBgl1A was from the GH1 family.

### Overexpression and Purification of Recombinant β-Glucosidases

**FIGURE 1:**
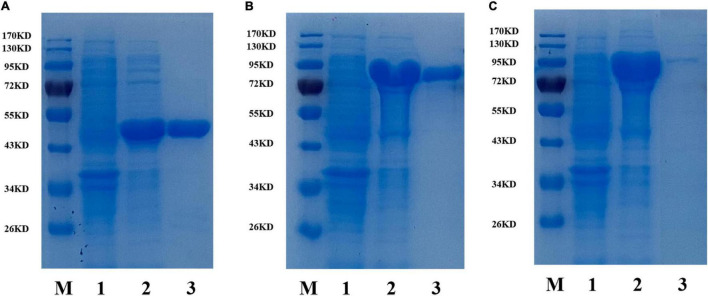
SDS-PAGE analysis of recombinant BaBgl1A **(A)**, BaBgl3A **(B)**, and BaBgl3B **(C)**. Lane M, protein markers; lane 1, culture lysate of recombinant β-glucosidases before isopropyl β-D-1-thiogalactopyranoside (IPTG) induction; lane 2, culture lysate of recombinant β-glucosidases after IPTG induction; lane 3, recombinant β-glucosidases purified from Ni sepharose fast-flow column.

To investigate the enzymatic properties of the three β-glucosidases, the enzymes were heterologously expressed in *E. coli* BL21 (DE3) with high enzyme production efficiency. The recombinant β-glucosidases were expressed as soluble proteins in *E. coli* BL21 (DE3) and showed single bands after purification using a Ni^2+^ affinity column of approximately 44, 81, and 88 kDa, respectively, which were close to their theoretical molecular mass (Figure 1). To compare the expression of the three enzymes, enzyme activity was determined by measuring the increase in absorbance of the reaction mixture at 405 nm using *p*NPβGlc as the substrate (Table 2). BaBgl1A was purified 2.3-fold, with 85.1% yield and 71.2 U/mg specific activity; BaBgl3A was purified 1.2-fold, with 9.2% yield and 3.9 U/mg specific activity; and BaBgl3B was purified 1.3-fold, with 14.1% yield and 7.6 U/mg specific activity. Based on the results of purification, it could concluded that the properties of BaBgl1A were better than those of BaBgl3A and BaBgl3B.

**TABLE 2:** Summary of the purification of recombinant three β-glucosidases from *Bifidobacterium adolescentis*.

Purification step		Volume (ml)	Total protein (mg)^a^	Activity (U)^b^	Specific activity (U/mg)	Purification (fold)	Yield (%)
BaBgl1A	Crude enzyme extract	18	21.6	664.8	30.8	1.0	100
	Ni sepharose fast-flow column	14	8.0	565.9	71.2	2.3	85.1
BaBgl3A	Crude enzyme extract	14	29.1	42.1	1.5	1.0	100
	Ni sepharose fast-flow column	7	2.2	3.9	1.8	1.2	9.2
BaBgl3B	Crude enzyme extract	13	25.5	53.7	2.1	1.0	100
	Ni sepharose fast-flow column	10.5	2.8	7.6	2.7	1.3	14.1

*^a^Protein was quantified according to the Bradford method using bovine serum albumin as standard.*

*^b^The activity was reported as activity on pNPβGlc.*

### Biochemical Characterization of Recombinant β-Glucosidases

**FIGURE 2:**
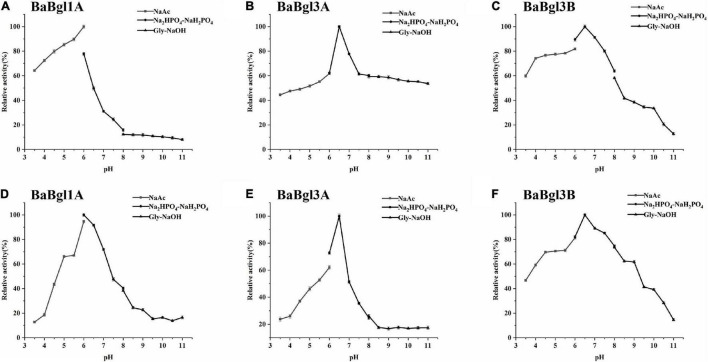
pH profiles of the purified recombinant enzymes (BaBgl1A, BaBgl3A, and BaBgl3B). The effects of pH and stability on BaBgl1A are shown in **(A,D)**. The effects of pH and stability on BaBgl3A are shown in **(B,E)**. The effects of pH and stability on BaBgl3B are shown in **(C,F)**. All tests of pH stability were performed at 4°C for 12 h. Values are mean ± SD from three biological replicates.

**FIGURE 3:**
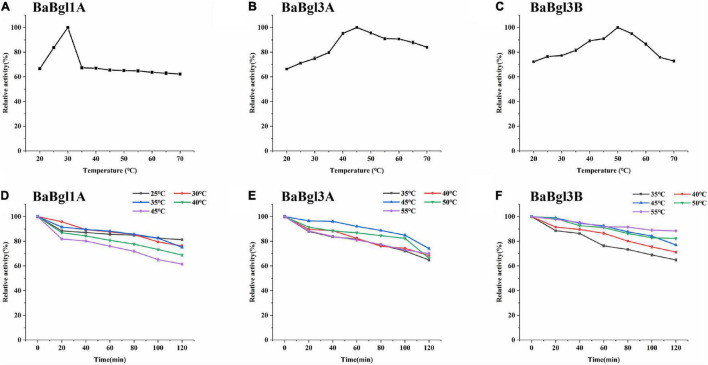
Temperature profiles of the purified recombinant enzymes (BaBgl1A, BaBgl3A, and BaBgl3B). The effects of temperature and stability on BaBgl1A are shown in **(A,D)**. The effects of temperature and stability on BaBgl3A are shown in **(B,E)**. The effects of temperature and stability on BaBgl3B are shown in **(C,F)**. BaBgl1A was tested in the buffer of 50 mM NaAc (pH 6.0). BaBgl3A and BaBgl3B were tested in the buffer of 50 mM Na_2_HPO_4_–NaH_2_PO_4_ (pH 6.5). Values are mean ± SD from three biological replicates.

The biochemical properties of the three β-glucosidases were examined from pH 3.5 to 11.0 with *p*NPβGlc as the substrate (Figure 2). The maximum activity of BaBgl1A, BaBgl3A, and BaBgl3B was observed at pH 6.0, 6.5, and 6.5, respectively. BaBgl1A and BaBgl3B were more stable than BaBgl3A. After pre-incubation at 4°C for 24 h, BaBgl1A had over 60% of the enzyme activity retrieved at pH 4.5–7.0, and BaBgl3B had over 60% of the enzyme activity retrieved at pH 4.0–8.0 (Figures 2D,F). The effect of temperature on enzyme activity was investigated at optimal pH. As shown in Figure 3, maximum activities of BaBgl1A, BaBgl3A, and BaBgl3B were observed at 30, 45, and 50°C, respectively. According to optimal temperature, the temperature stability of BaBgl1A was investigated by varying the temperature from 25 to 45°C at optimal pH and those of BaBgl3A and BaBgl3B were investigated by varying the temperature from 35 to 55°C (Figures 3A–C). The results showed that BaBgl3A exhibited poor thermal stability. More than 80% of its activity remained after treatment at over 35°C for 1 h; however, the enzyme activity decreased below 80% after incubation at all the temperature ranges for 2 h (Figure 3E). BaBgl1A and BaBgl3B had better thermal stability than BaBgl3A, as their enzyme activity remained over 80% at a wider temperature range (Figures 3D,F). This indicated that the optimal conditions for BaBgl1A, BaBgl3A, and BaBgl3B were pH 6.0 and 30°C, pH 6.5 and 45°C, and pH 6.5 and 50°C, respectively.

### Effect of Additives on Enzymatic Activity

**FIGURE 4:**
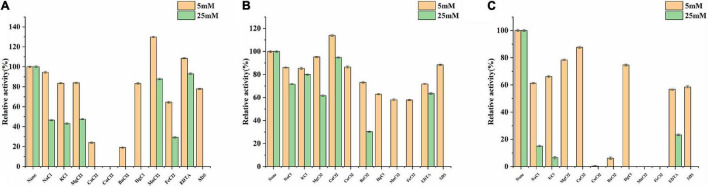
Effects of metal ions and reagents on BaBgl1A, BaBgl3A, and BaBgl3B activity. **(A)** Effects of metal ions and reagents (5 and 25 mM) on BaBgl1A activity. **(B)** Effects of metal ions and reagents (5 and 25 mM) on BaBgl3A activity. **(C)** Effects of metal ions and reagents (5 and 25 mM) on BaBgl3B activity. Values are mean ± SD from three biological replicates.

For determining the efficiency of hydrolysis, the effects of metal ions and reagents on BaBgl3A, BaBgl1A, and BaBgl3B activity were further investigated. As shown in Figure 4, at the concentration of 5 mM, only Mn^2+^ and EDTA slightly activated BaBgl1A, and Ca^2+^ slightly activated BaBgl3A. In contrast, Ca^2+^, Cu^2+^, and Ba^2+^ significantly inhibited BaBgl1A and BaBgl3B. At the concentration of 50 mM, all the metal ions and reagents used in this study had little or inhibitory effect on the activities of the three enzymes.

**FIGURE 5:**
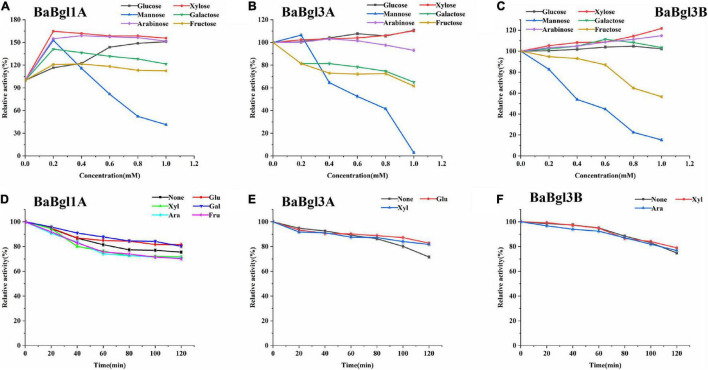
Effects of sugar and stability on BaBgl1A, BaBgl3A, and BaBgl3B activity. The effects of sugar and stability on BaBgl1A are shown in **(A,D)**. The effects of sugar and stability on BaBgl3A are shown in **(B,E)**. The effects of sugar and stability on BaBgl3B are shown in **(C,F)**. Values are mean ± SD from three biological replicates.

Furthermore, different monosaccharides were added to the reaction system to investigate the effect of polyols on the three β-glucosidases. Most monosaccharides could increase BaBgl1A and BaBgl3B activity, while mannose and fructose acted as competitive inhibitors for BaBgl1A and BaBgl3B. Mannose could significantly inhibit their activity by more than 70% at 1-M monosaccharide concentration (Figures 5A,C). Glucose and xylose could increase the activity of only BaBgl3A by 10% at 1-M monosaccharide concentration (Figure 5B). The residual activities of the enzymes after incubation at optimal conditions for 120 min were evaluated. As shown in Figure 5D, the addition of glucose and galactose to the reaction resulted in a stronger protection effect of enzyme activity, and BaBgl1A still retained more than 80% vitality. Similar results could be seen in Figures 5E,F; compared to that observed in the blank control, glucose and xylose could increase the residual activity of BaBgl3A, while xylose and arabinose could increase the residual activity of BaBgl3B. The results mentioned above indicate that glucose, xylose, and galactose can be used as candidates for β-glucosidase activity enhancers in *B. adolescentis.*

### Substrate Specificity of Recombinant β-Glucosidases

The activities of the three recombinant enzymes from *B. adolescentis* were assessed using aryl-glycosides and disaccharides. The results (Table 3) showed that BaBgl1A, BaBgl3A, and BaBgl3B exhibited the best activity toward *p*NPβGlc when aryl-glycosides were used as substrates. In addition, BaBgl3A was also active on *p*NPβXyl and *p*NPαAra*p*, and BaBgl3B was also active on *p*NPβGal, *p*NPβXyl, and *p*NPαAra*p*. These results indicated that BaBgl1A had better substrate specificity than BaBgl3A and BaBgl3B. When disaccharides were used as substrates, BaBgl1A, a GH1 enzyme, showed good activity on sophorose [glucose-β-(1→2)-glucose] and medium activity on laminaribiose [glucose-β-(1→3)-glucose] and cellobiose [glucose-β-(1→4)-glucose], while there was no activity on gentiobiose [glucose-β-(1→6)-glucose]. The linkage preference of BaBgl1A for disaccharides was β-1, 2 > β-1, 3 > β-1, 6 > > > β-1, 4. The GH3 enzymes BaBgl3A and BaBgl3B exhibited good activity on laminaribiose [glucose-β-(1→3)-glucose] and cellobiose [glucose-β-(1→4)-glucose], respectively. They also showed a different activity on other disaccharides. Therefore, the disaccharide substrate selectivity of the three β-glucosidases from *B. adolescentis* differed. BaBgl1A might mainly act as β-1,2-glucosidase, BaBgl3A might mainly act as β-1,3-glucosidase, and BaBgl3A might mainly act as β-1,4-glucosidase.

**TABLE 3:** Relative activity of β-glucosidases on different aryl-glycosides and disaccharides.

Substrate	Relative activity (%)		
	
	BaBgl1A	BaBgl3A	BaBgl3B
*p*NPβGlc	100	100	100
*p*NPβGal	7.60 ± 0.36	12.45 ± 1.24	31.02 ± 0.82
*p*NPβXyl	2.14 ± 0.02	51.45 ± 1.90	52.24 ± 0.41
*p*NPαGlc	1.59 ± 0.01	7.47 ± 1.25	4.90 ± 1.23
*p*NPαGal	0.97 ± 0.06	17.84 ± 0.83	12.24 ± 2.45
*p*NPαXyl	1.22 ± 0.06	23.24 ± 2.08	22.04 ± 1.64
*p*NPαAra*f*	2.66 ± 0.13	0.83 ± 1.87	–
*p*NPαAra*p*	2.85 ± 0.07	64.73 ± 1.24	44.08 ± 1.23
Sophorose	100.00	20.83	–
Laminaribiose	46.55	100.00	23.98
Cellobiose	29.83	52.78	100.00
Gentiobiose	9.65	63.19	66.84

**FIGURE 6:**
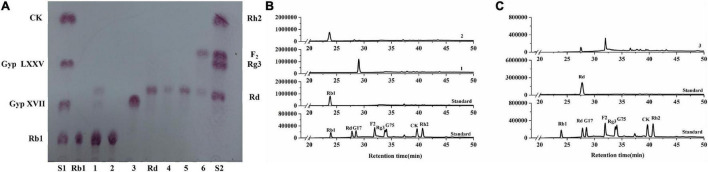
Thin-layer chromatography **(A)** and high-performance liquid chromatography **(B,C)** analyses of the degradation products of Rb1 **(B)** and Rd **(C)** by BaBgl1A, BaBgl3A, and BaBgl3B. The degradation of Rb1 by BaBgl1A is shown in (A3 B1). The degradation of Rb1 by BaBgl3A is shown in (A1 B2). The degradation of Rb1 by BaBgl3B is shown in (A2). The degradation of Rd by BaBgl1A is shown in (A6 C3). The degradation of Rd by BaBgl3A is shown in (A4). The degradation of Rd by BaBgl3B is shown in (A5). The reaction was incubated with recombinant enzymes at 37°C for 2 h.

To further verify the substrate selectivity of the three recombinant β-glucosidases, ginsenosides Rb1 and Rd, which contain different glucose linkage types (β-glucose, β-1,2-glucose, and β-1,6-glucose), were used as the substrates. The results of TLC and HPLC revealed that, in the presence of BaBgl1A and BaBgl3A, ginsenoside Rb1 was converted into different metabolites, while BaBgl3B could not hydrolyze the ginsenosides, indicating that BaBgl3A and BaBgl1A are ginsenoside-hydrolyzing enzymes (Figure 6A). To verify the bioconversion pathway and efficiency of BaBgl3A and BaBgl1A, individual ginsenosides were used as substrates for the biotransformation, and the products were analyzed using HPLC. As shown in Figure 6B, Rb1 could be converted into Rd and Gyp XVII by BaBgl3A, with yields of 9.65 and 5.65%, respectively. This suggested that BaBgl3A can remove the outer glucose linked to the β-1,6 linkage of the C-20 position and that linked to the β-1,2 linkage of the C-3 position in ginsenoside Rb1, while it had no activity toward Rd (Figures 6B,C). This indicated that BaBgl3A had weak activity toward the β-1,2-/β-1,6 linkage of glucose. Similarly, BaBgl1A could hydrolyze the β-1,2 linkage of the C-3 position in ginsenosides Rb1 and Rd, producing the minor ginsenoside Gyp XVII and F_2_ with yields of 100 and 72.16%, respectively (Figures 6B,C). The transformation was almost completed in 2 h, which suggested that BaBgl1A had strong activity toward the β-1,2 linkage of glucose.

## Discussion

Previously, certain β-glucosidases from *Bifidobacterium* have been expressed as recombinant proteins in *E. coli*, most of which belong to the GH3 family (Matsumoto et al., 2015; Guadamuro et al., 2017; Florindo et al., 2018). All these proteins exhibited medium activity toward aryl-glycosides other than *p*NPβGlc. In this study, although three β-glucosidase genes from *B. adolescentis* were overexpressed in *E. coli* BL21 (DE3), their activities toward different substrates varied. The recombinant β-glucosidases BaBgl3A and BaBgl3B showed sequence similarity to the GH3 family, while the sequence similarity between BaBgl3A and BaBgl3B was only 32%, which might lead them to exhibit different multi-functional glycoside hydrolase activities. BaBgl1A, belonging to the GH1 family, exhibited a high degree of specificity toward *p*NPβGlc. This was consistent with that observed for the GH1 β-glucosidase from the marine bacterium *Alteromonas* sp. L82 (Sun et al., 2018). In addition, the enzymatic activity of BaBgl1A was 71.2 U/mg, which was higher than those of the other two enzymes, and the recovery of BaBgl3A and BaBgl3B was also lower than that of BaBgl1A. This might be because they belong to different glycoside hydrolase families, and hence their amino acid sequences varied.

The optimum reaction condition of an enzyme affects the enzyme ratios, which play an important role in its function (Elena et al., 1994). In this study, the three recombinant β-glucosidases were active at an optimal pH of around pH 7.0, which might be because these β-glucosidases are from the human gut microbiota. Studies have shown that many glycosides from the human gut microbiota were active at pH 7.0, such as β-xylosidase from *Bacteroides ovatus* strain ATCC 8483 (Zhou et al., 2019), α-glucosidases from *Bacteroides thetaiotaomicron* (Chaudet et al., 2012), and endo-1,6-β-glucanase from *B. thetaiotaomicron* (Temple et al., 2017). In contrast, the optimal temperature required for the activity of these three enzymes spanned a large range, from 30 to 50°C. Similar studies have shown that the optimal temperature for the activity of α-glucosidase from *B. longum* KCTC 3127 was 75°C (Kim et al., 2017), and the 1,4-α-glucan branching enzyme from *B. longum* subsp. Longum ATCC 55813 was optimally active at 25°C (Dan et al., 2020). Most metal ions and reagents negatively affected the three β-glucosidases. Only Mn^2+^ and Ca^2+^ slightly activated the enzymatic activities of BaBgl1A and BaBgl3A, respectively, which might be because they could bind to the active site of the enzyme and increase its stability (Xie et al., 2015). In addition, different monosaccharides, including glucose, galactose, and xylose, exerted positive effects on the recombinant enzymes. A similar study has shown that the activity of β-glucosidase from *Thermotoga petrophila*, TpBgl3, increased when incubated with galactose, xylose, and arabinose (Cota et al., 2015). Research had shown that sugar could strengthen the interaction between protein core hydrophobic residues and lead to the overall increase of protein stability (Padma and Laxmi, 2008). The addition of glucose, galactose, and xylose might stabilize the protein structure of those β-glucosidases. The other reason might be the direct interactions of those monosaccharides and β-glucosidases in the binding pocket, resulting in the increase of protein catalytic activity.

**FIGURE 7:**
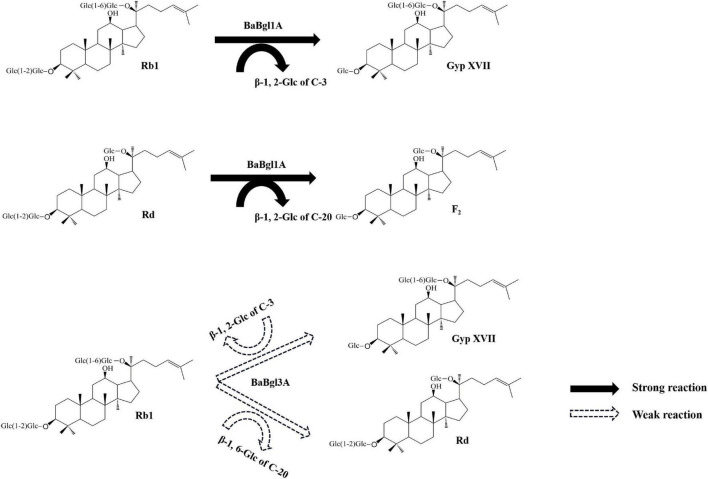
Biotransformation pathways of ginsenosides Rb1 and Rd by recombinant BaBgl1A and BaBgl3A.

Previously, certain ginsenoside-hydrolyzing β-glucosidases have been heterologously expressed in *E. coli*, such as β-glucosidases from *Bifidobacterium longum* H-1 (Jung et al., 2012), *Thermotoga thermarum* (Zhao et al., 2013), and *Cellulosimicrobium cellulans* sp. 21 (Yuan et al., 2015). The hydrolysis behavior of BaBgl1A on ginenosides was similar to those of β-glucosidases, which could transform Rb1 into Rd effectively. The analysis of these enzymes revealed that their amino acid sequences differed and that their optimal temperature and pH varied, although their molecular weights were similar, and all were specific for aryl-glycosides and belonged to the GH1 family. These properties may result in their ability to hydrolyze the glucose at the C-3 positions in ginsenosides Rb1 and Rd. Compared to BaBgl1A, BaBgl3A, and BaBgl3B exhibited poor selectivity toward ginsenosides. The BaBgl3A could hydrolyze the sugar moieties at both the C-3 and C-20 positions in the PPD-type ginsenoside Rb1. A similar result has been found in a study on β-glucosidase from *Microbacterium esteraromaticum* (Quan et al., 2012), which could transform Rb1 into C-K. BaBgl3A might completely transform Rb1 into C-K if the amount of enzyme and transformation time was sufficient. Although the amino acid sequences, molecular weight, and multi-substrate selectivity of BaBgl3A and BaBgl3B were similar, BaBgl3B was not active toward ginsenosides, which might be because it was mainly a β-1,3-glucosidase, which cannot hydrolyze β-1,2-glucose or β-1,6-glucose in ginsenosides. Based on the ginsenoside transformation products obtained, we have proposed a model for the transformation of Rb1 and Rd by BaBgl1A and BaBgl3A (Figure 7). It can be speculated that BaBgl1A had higher ginsenoside conversion capacity and substrate specificity than the others.

## Conclusion

In conclusion, three β-glucosidase genes from *B. adolescentis* here were cloned and expressed. The biochemical characterization and selectivity of these three β-glucosidases (BaBgl1A, BaBgl3A, and BaBgl3B) were compared and analyzed. BaBgl1A was found to be an exclusive β-1,2-glucosidase, which transformed ginsenosides Rb1 and Rd to minor ginsenosides Gyp XVII and ginsenoside F_2_; BaBgl3A acted as β-1,3-glucosidase, with negligible specificity for the β-1,2/β-1,6-glucosidic linkage in ginsenoside Rb1. BaBgl3B acted as β-1,4-glucosidase, which was not active toward ginsenoside Rb1 and Rd. The different specific β-glucosidases may be used as effective biocatalytic tools for the further development of *B. adolescentis* as a model system as well as for understanding the molecular mechanism of oligosaccharide degradation by probiotics.

## Data Availability Statement

The original contributions presented in the study are included in the article/Supplementary Material, further inquiries can be directed to the corresponding authors.

## Author Contributions

YH and JZ conceived and designed the experiments. YH, LZ, HH, and ZS performed the experiments and analyzed the data. YH, LZ, JZ, and DL wrote and edited the manuscript. All authors reviewed the manuscript, read, and approved the final manuscript.

## Conflict of Interest

The authors declare that the research was conducted in the absence of any commercial or financial relationships that could be construed as a potential conflict of interest.

## Publisher’s Note

All claims expressed in this article are solely those of the authors and do not necessarily represent those of their affiliated organizations, or those of the publisher, the editors and the reviewers. Any product that may be evaluated in this article, or claim that may be made by its manufacturer, is not guaranteed or endorsed by the publisher.

## Footnotes

1
http://www.cazy.org/
