# Supplementary material for: Study on the Biochemical Characterization and Selectivity of Three β-Glucosidases From Bifidobacterium adolescentis ATCC15703
Source: Front Microbiol. 2022 Apr 8;13:860014. doi: 10.3389/fmicb.2022.860014 (PMC9024363; doi:10.3389/fmicb.2022.860014)
Supplement: Supplementary file 1 [file Data_Sheet_1.docx]

***Supplementary Material***

**Supplementary Figure 1**

**
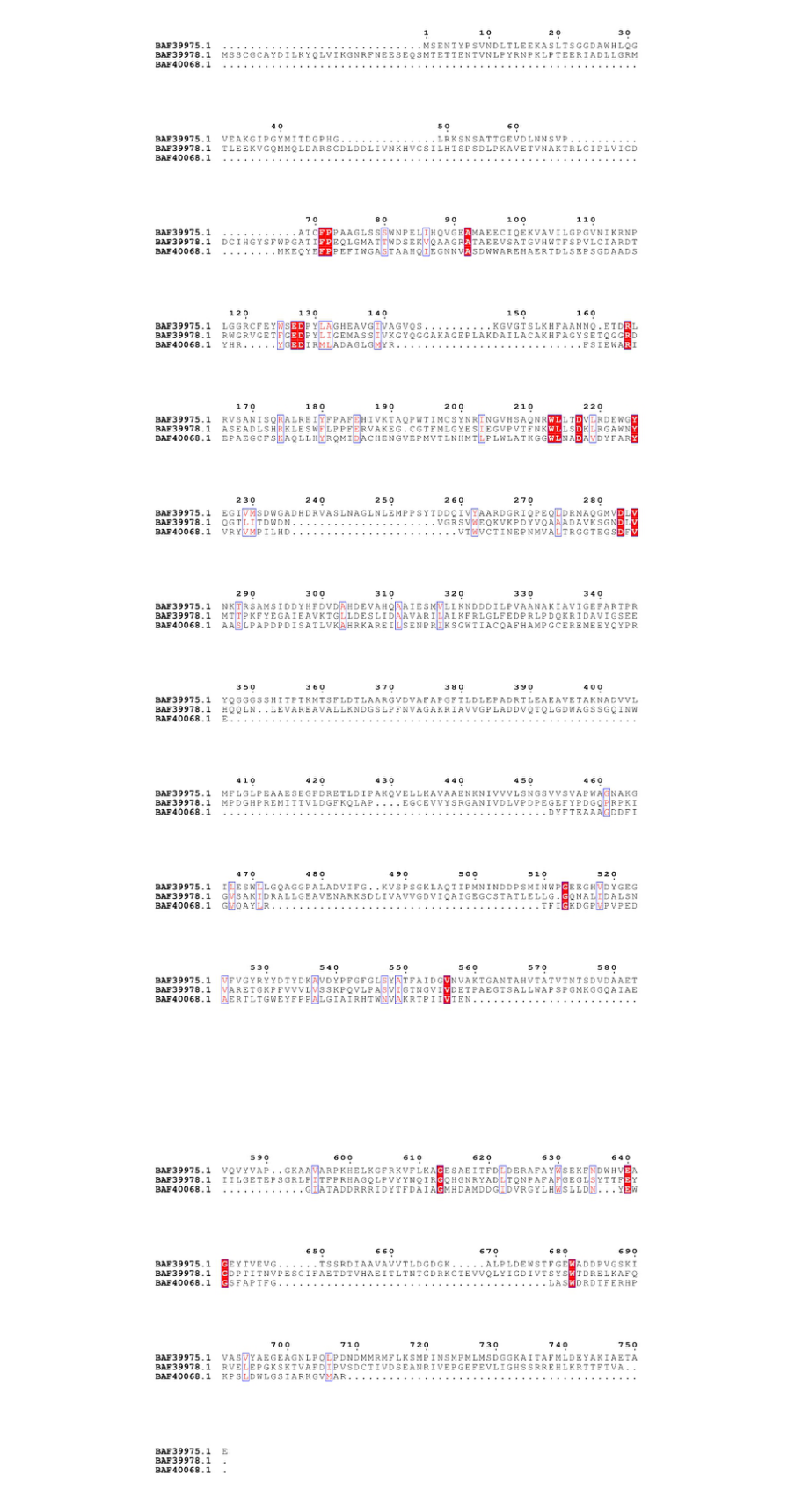
**

**Supplementary Figure 1.**Sequence alignment analysis of recombinantβ-glucosidases from *Bifidobacterium adolescentis* ATCC15703. (The genebankaccessions of BaBgl1A, BaBgl3AandBaBgl3B wereBAF40068.1, BAF39975.1 and BAF39978.1, respectively)

**
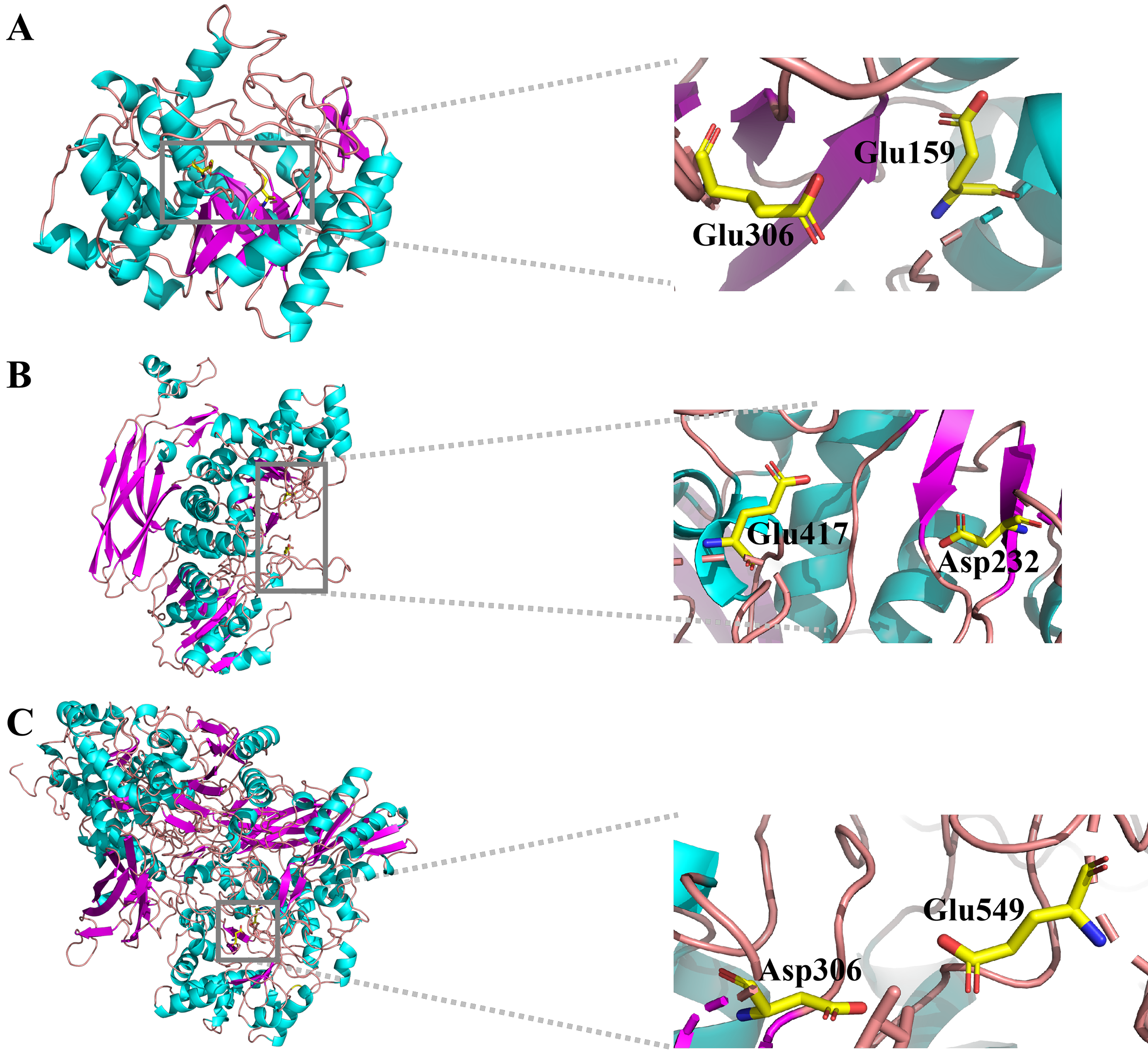
**

**Supplementary Figure 2.** 3D structural models of BaBgl1A (A), BaBgl3A(B), and BaBgl3B (C) were generated using Pymol. Parallel β-strands, linkers and α-helices are shown in cyan, orange andmagenta respectively. Glu (159-BaBgl1A, 306-BaBgl1A, 417-BaBgl3A and 549-BaBgl3B), and Asp (232-BaBgl3A and 306-BaBgl3B)residues are shown in yellow lines.
